# Effects of Dietary Supplementation of a Microalgae Extract Containing Fucoxanthin Combined with Guarana on Cognitive Function and Gaming Performance

**DOI:** 10.3390/nu15081918

**Published:** 2023-04-15

**Authors:** Megan Leonard, Jonathan Maury, Broderick Dickerson, Drew E. Gonzalez, Jacob Kendra, Victoria Jenkins, Kay Nottingham, Choongsung Yoo, Dante Xing, Joungbo Ko, Rémi Pradelles, Mark Faries, Wesley Kephart, Ryan Sowinski, Christopher J. Rasmussen, Richard B. Kreider

**Affiliations:** 1 Exercise & Sport Nutrition Lab, Department of Kinesiology and Sports Management, Texas A&M University, College Station, TX 77843, USA; meganleonard10@tamu.edu (M.L.); dickersobl5@email.tamu.edu (B.D.); dg18@tamu.edu (D.E.G.); jkendra@tamu.edu (J.K.); victoria.jenkins@tamu.edu (V.J.); kvnottingham@tamu.edu (K.N.); choongsungyoo@tamu.edu (C.Y.); dantexing@tamu.edu (D.X.); joungboko10@tamu.edu (J.K.); mark.faries@ag.tamu.edu (M.F.); rjs370@tamu.edu (R.S.); crasmussen@tamu.edu (C.J.R.); 2 Microphyt, Research & Development Department, 34670 Baillargues, France; jonathan.maury@microphyt.eu (J.M.); remi.pradelles@microphyt.eu (R.P.); 3 Texas A&M AgriLife Extension, Texas A&M University, College Station, TX 77843, USA; 4 Department of Kinesiology, University of Wisconsin, Whitewater, WI 53190, USA

**Keywords:** nootropic, fucoxanthin caffeine, ergogenic aid, sports nutrition, esports, gaming

## Abstract

Background: Esports competitive gaming requires selective visual attention, memory, quick judgment, and an ability to sustain psychomotor performance over time. Fucoxanthin is a carotenoid, found in specific microalgae varieties such as *Phaeodactylum tricornutum* (*PT*), that has been purported to possess nootropic and neuroprotective effects through its anti-inflammatory and antioxidant properties. This study evaluated whether acute and 30-day supplementation of an extract of *PT* from microalgae combined with guarana (a natural source of caffeine) affects cognitive function in gamers. Materials and Methods: In a double-blind, placebo-controlled manner, 61 experienced gamers (21.7 ± 4.1 years, 73 ± 13 kg) were randomly assigned to ingest a placebo (PL), a low-dose (LD) supplement containing 440 mg of *PT* extract including 1% fucoxanthin +500 mg of guarana containing 40–44 mg caffeine (MicroPhyt™, Microphyt, Baillargues, FR), or a high-dose (HD) supplement containing 880 mg of *PT* extract +500 mg of guarana for 30 days. At baseline, cognitive function tests were administered before supplementation, 15 min post-supplementation, and after 60 min of competitive gameplay with participants’ most played video game. Participants continued supplementation for 30 days and then repeated pre-supplementation and post-gaming cognitive function tests. General linear model univariate analyses with repeated measures and changes from baseline with 95% confidence intervals were used to analyze data. Results: There was some evidence that acute and 30-day ingestion of the *PT* extract from microalgae with guarana improved reaction times, reasoning, learning, executive control, attention shifting (cognitive flexibility), and impulsiveness. While some effects were seen after acute ingestion, the greatest impact appeared after 30 days of supplementation, with some benefits seen in the LD and HD groups. Moreover, there was evidence that both doses of the *PT* extract from microalgae with guarana may support mood state after acute and 30-day supplementation. Registered clinical trial #NCT04851899.

## 1. Introduction

Considering the rapid development and growth of esports competitions globally and applications in occupational and military activities [1], it is understandable that there is interest in identifying ways to improve gaming performance. Research has shown that gamers exhibit stronger cognitive functions (e.g., executive function, visual selective attention, cognitive flexibility, task switching) than those who do not play video games [2,3,4,5,6]. Competitive gaming typically involves playing several rounds of games a day and/or different games over several days of competition. Therefore, gamers must maintain high energy from session to session and during longer gaming sessions to be competitive. To this end, gamers often consume caffeinated beverages and/or energy drinks containing various natural ingredients to help them stay alert [7,8]. The International Society of Sports Nutrition (ISSN) indicated in its position stands that the primary ergogenic nutrient in energy drinks and shots is caffeine [9,10]. Guarana (*Paulliniacupana*) is a natural caffeine source commonly found in popular energy drinks [9,11,12,13,14]. Scholey et al. [13] reported that guarana might optimize the benefits of the antioxidant multivitamin complex on cognitive performance in healthy populations. However, the ISSN and others also indicated more research was needed to determine whether adding other purported nootropic nutrients to caffeinated drinks and supplements would provide additional benefits [9,10,14].

Marine environments harbor vast biologically diverse microalgae populations representing a large source of bioactive molecules, such as carotenoids, pigments, fatty acids, peptides, and sterols [15,16,17]. Fucoxanthin is a carotenoid found in microalgae. It is known for its neuroprotective effects through anti-inflammatory and antioxidant actions on different signaling pathways (e.g., Nrf-ARE and Nrf2-autophagy) and its ability to bypass the blood–brain barrier [18,19,20]. Wang and colleagues [21] reported that fucoxanthin and omega-3 long-chain polyunsaturated fatty acids (*n*-3 LC-PUFAs), another nutrient reported to improve cognitive function and/or health, can be produced simultaneously in specific microalgae varieties such as the diatom *Phaeodactylum tricornutum* (PT). Of the LC-PUFAs, eicosapentaenoic acid (EPA) is an agonist of peroxisome proliferator-activated receptor alpha (PPARα), which may activate or inhibit several signaling pathways (e.g., decrease neuroinflammation and oxidative stress, autophagy, lipid metabolism) directly contributing to neuroprotection [22]. Phycoprostans, oxylipins of microalgal origin, and other compounds found specifically in *PT* microalgae extract (technical name: PhaeoSol) act on the mitochondria of neurons by decreasing the membrane potential, which has the effect of reducing reactive oxygen species (ROS) production [23]. Combining these different molecules into a single *PT* microalgae extract may have the potential to improve cognitive performance.

The effects of *PT* microalgae extract on cognitive function parameters were evaluated in a proof-of-concept pre-clinical study using a specific mouse model of accelerated aging (unpublished data in preparation for publication). Preliminary results showed a significant improvement in short-term and spatial working memory and markers of brain oxidative and inflammatory stress at low doses (120 mg/day as an equivalent daily human dose). Interestingly, *PT* microalgae extract was found more effective than Ginkgo biloba extract for all doses tested, a known reference extract for improving brain function [24]. Based on these data and literature analysis, co-ingesting guarana with this microalgae-based extract of *PT* ingredient might enhance cognitive function in populations such as competitive video gamers. To our knowledge, the only nutritional strategies that have been studied to assess the potential ergogenic value in experienced gamers include inositol bonded arginine silicate [25,26,27], a natural source of caffeine with polyphenolic antioxidants [28], and a caffeinated energy drink containing several nutrients purported to affect cognitive function [8]. Obviously, more research is needed to determine whether various nutritional interventions may impact gaming performance. This study evaluated whether acute and 30-day supplementation of microalgae extract from *PT* with a natural source of caffeine (guarana) affects cognitive function and gaming performance in experienced gamers. If so, these microalgae extract from PT may serve as a novel nootropic ingredient to add to caffeinated pre-workout supplements, energy drinks, and/or supplements for gamers and related populations.

## 2. Methods

### 2.1. Design of Study

**Figure 1:**
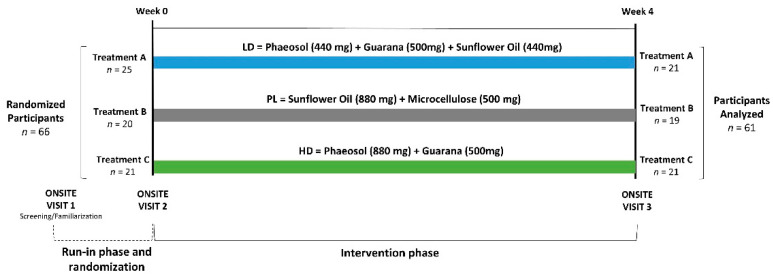
Overview of experiment study timeline for the placebo (PL), low-dose (LD), and high-dose (HD) groups.

This study was performed as a placebo-controlled, double-blind, parallel-arm intervention trial at a university clinical research facility (see Figure 1). Nutritional supplementation served as the independent variable. Primary dependent variable outcomes included a change from baseline to 4 weeks on cognitive function. Secondary endpoints were changes in performance on the light tracking reaction test, gaming scores, stimulant sensitivity and side effect questionnaires, mood state, fatigue, ratings of eye irritability, and sleep quality index.

### 2.2. Study Participants

This investigation complied with the Declaration of Helsinki ethical standards for performing human participant research and was approved by the university’s Institutional Review Board (IRB2021-0219) and registered with clinicaltrials.gov (NCT04851899). Experienced gamers (18–40 years, 18–34.9 kg/m^2^ body mass index) were recruited for this study. Inclusion criteria were (1) self-reported history of playing video games for 5 or more hours a week for at least 6 months prior to screening; (2) agreement to use their operator-oriented action or strategy video game that they had played at least 21 times over the last 3 months; (3) no recent ingestion (<2 weeks) of a dietary supplement that affects cognitive function; (4) give written informed consent and agree to consume the investigational product daily during the study; (5) free living (living in a private home, alone or with family, and able to maintain their health and hygiene without assistance); (6) willing to maintain consistent sleep habits the evening before study visits; and (7) agree to continue their patterned use of the game between study visits.

Interested volunteers were screened by phone to determine preliminary eligibility. Prospective participants meeting screening criteria were scheduled for a familiarization session where the study design and testing procedures were explained and they signed informed consent statements, completed a medical history, and had a physical examination performed to assess qualifications. Participants were excluded if they (1) consumed caffeine and alcohol within 12 h prior to each study visit; (2) consumed dietary supplements that may affect cognition and/or have a stimulant effect (e.g., guarana, cocoa, ginseng, bacopa, ginkgo biloba, guayusa, yerba mate, energy drinks, other products containing fucoxanthin) at least 7 days before each testing session; (3) were pregnant, breastfeeding, or had a desire to become pregnant during the study; (4) had an untreated psychotic or major depressive disorder or a history of cognitive deficit; (5) had uncontrolled hypertension/diabetes/thyroid/heart disease, cancer etc.); (6) had significant neurological disease; (7) had planned major changes in lifestyle (i.e., diet, dieting, exercise level, travelling) during the study; (8) had a history within previous 12 months of alcohol or substance abuse; (9) had a known allergy to any of the ingredients in the supplement; and/or (10) were not willing to supply their own gaming system and/or game.

**Figure 2:**
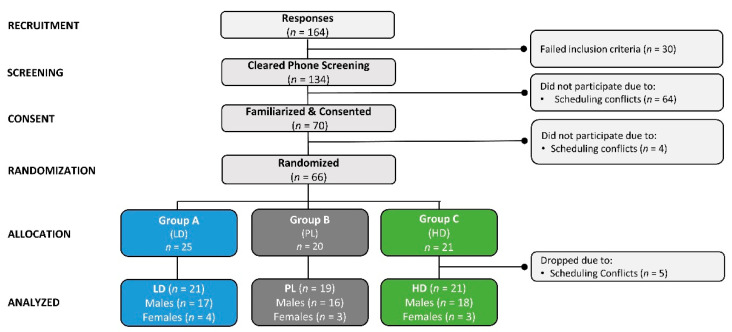
Consolidated Standards of Reporting Trials (CONSORT) chart for the placebo (PL), low-dose (LD), and high-dose (HD) groups.

Figure 2 shows a Consolidated Standards of Reporting Trials (CONSORT) diagram. A total of 164 individuals responded to study advertisements and were assessed for eligibility, with 134 meeting phone screening criteria. Due to scheduling conflicts and delays in starting the study, 70 men and women were familiarized and consented to participate. Of these, 66 individuals were randomly assigned into groups. Five participants were assigned to groups but decided not to participate before testing. A total of 25, 20, and 21 participants were allocated to groups A (low-dose *PT* microalgae extract + guarana (LD)), B (placebo (PL)), or C (high-dose *PT* microalgae extract + guarana, (HD)), respectively. After testing began, five participants withdrew from the study due to scheduling conflicts. Therefore, 61 participants (51 men and 10 women) completed the study.

### 2.3. Testing Protocol

**Figure 3:**
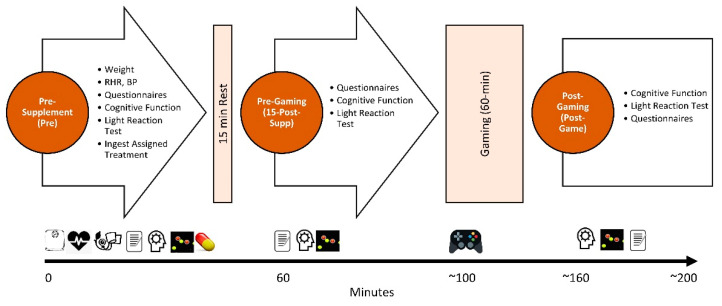
Timeline of testing sessions. RHR represents resting heart rate, while BP represents blood pressure. Questionnaires included the Profile of Mood States, fatigue and eye irritability rating, side effects, and sleep quality. Cognitive function tests included the Berg-Wisconsin Card Sorting, Go/No-Go, Sternberg, and Psychomotor Vigilance Task tests.

Figure 3 overviews the timeline for each experiment. Participants attended a familiarization session and completed two experimental testing sessions (baseline and week 4). At the familiarization, participants completed health history questionnaires and underwent a general physical exam which included determining height, weight, resting heart rate, and blood pressure. They were also informed about the general methods of the study and performed the cognitive function and light reaction tests several times to establish testing reliability. Those eligible to participate were scheduled for a baseline visit that included practicing the study assessments (i.e., cognitive function and light reaction tests). Participants recorded food and fluid intake for 4 days before testing, refrained from consuming atypical amounts of caffeine and other stimulants not normally consumed in their diet for 48 h, and fasted for 12 h before each testing session. The video game type (strategy or action based) was confirmed during this visit. Each participant was responsible for bringing their preferred video game and gaming platform with accessories. Participants agreed to bring and play the same video game for both visits and to maintain their normal frequency of playing the game between visits to minimize learning curve bias. All scores of the chosen game before dosing were recorded through a photo or screenshot.

Participants were weighed, completed pre-supplementation questionnaires, rated eye fatigue and irritability using a visual analog scale (VAS), and performed a battery of cognitive function tests described below. The light reaction test was then performed, in which a gaming mouse was used to track and identify objects. Participants were then randomly assigned to ingest a placebo or one of the two study supplements (Pre). Fifteen minutes following ingestion (15-Post-Supp), participants repeated all tests. Participants then played their video game for 60 min competitively. Immediately afterward, the participants completed the cognitive tests, light reaction tests, and questionnaires (Post-Game). Participants were then given the appropriate amount of their assigned group supplement and instructed to take it daily for 30 days. Participants returned to the lab after 30 days of supplementation to repeat the Pre and Post-Game experiments while ingesting the remaining supplement.

### 2.4. Supplementation Protocol

In a double-blind and randomized manner, gamers ingested two soft gels + one capsule of either (1) PL consisting of 2 × 440 mg soft gels containing sunflower oil (Helianthus annuus seed oil, Ardex, Lavilledieu, France) + 1 × 500 mg capsule containing microcellulose oil (Vegetal, Greencaps, Teyran, France); (2) LD consisting of 1 × 440 mg *PT* microalgae extract soft gel (*PT* microalgae extract, Microphyt, Baillargues, France) + 1 × 440 mg soft gel of sunflower oil + 1 × 500 mg capsule of guarana (organic guarana extract containing 8–11% (40–55 mg) of native caffeine (*Paulliniacupana*, Greencaps, Teyran, France)); or (3) HD consisting of 2 × 440 mg *PT* microalgae extract soft gels + 1 × 500 mg guarana capsule. Placebo soft gels/capsules were similar in appearance and packaging for double-blind administration by the study sponsor. A manufacturer certificate of analysis documented supplement contents and purity. *Phaeodactylum tricornutum* microalgae extract is exclusively produced by the sponsor. Encapsulation in soft gels and packaging in blister packs was performed by Delpharm (Evreux, France) and Diephez (Selt, France), respectively. Dynveo (Dynveo, France) manufactured guarana capsules and the corresponding placebo under Good Manufacturing Practice (GMP) conditions and packaged them in labeled bottles for double-blind administration. The participants were asked to store blister packs in refrigerators (between +2 and +8 °C) and bottles at ambient temperature.

PhaoeSol is a branded microalgae-based nutritional ingredient developed with a patented industrial production process that has New Dietary Ingredient (NDI) status from the FDA (*PT* microalgae extract, Microphyt, Baillargues, France). For this study, we used an extract of the microalgae *PT*, standardized to 1.0% fucoxanthin content by adding a food-grade medium-chain triglyceride (MCT) from coconuts. Adding MCT is essential to obtain a paste that is not too viscous. Otherwise, homogenization is impossible, and the ethanolic evaporation (i.e., the last step of the production process) cannot be completed. *Phaeodactylum tricornutum* microalgae extract is intended for use as a source of the naturally occurring carotenoid fucoxanthin in food supplement products for the general population at levels not to exceed 440 mg/person/day for a maximal duration of 30 days (equivalent to 8.8 mg fucoxanthin/person/day) and 220 mg/person/day without duration limit of *PT* microalgae extract standardized to 2.0% of fucoxanthin. The participants took the assigned supplement at home, except on study visit days when they consumed the assigned product on-site.

## 3. Procedures

### 3.1. Demographics

Weight and height were obtained using a calibrated (±0.02 kg) electronic scale (Health-O-Meter Professional 500 KL, Pelstar LLC, Alsip, IL, USA). Seated resting heart rate and blood pressure were obtained after resting for 5 min. Cardiovascular hemodynamics were determined using a Connex^®^ ProBP™ 3400 digital blood pressure device (Welch Allyn, Tilburg, NL, USA) using standard procedures [29].

### 3.2. Diet Control

For diet consistency, participants recorded food and beverage intake for 4 days before the first testing session using a phone application (MyFitnessPal, Inc., Baltimore, MD, USA) or written food logs [30,31]. Participants replicated this diet before each testing session.

### 3.3. Cognitive Assessment

Cognitive function was assessed by having participants perform the Psychology Experiment Building Language (PEBL) cognitive test battery (Version 2.1, http://pebl.sourceforge.net; accessed on 14 October 2021) [32]. A detailed description of each test and methods employed for the PEBL test in our lab was previously described [26,33]. The assessment battery included the Berg-Wisconsin Card Sorting Task test (BCST) which uses reaction time and accuracy of sorting cards to evaluate reasoning, learning, executive function, attention shifting (i.e., flexibility in responding to changing reinforcement schedules), and impulsiveness [32,34,35,36,37]; the Go/No-Go Task test that assesses response control and sustained attention in responding (“Go”) or not responding (“No-Go”) to seeing the letters P or R appear on a computer monitor [34,35,38]; the Sternberg Task test (STT) that entails identifying visual stimuli as either present or absent within 2, 4, and 6 letter sequences in 3, 6, 9, 12, 15, or 18-second intervals to assess short-term/working memory involving cognitive control processes with the resulting reaction time and accuracy [34,35,39]; and the Psychomotor Vigilance Task test (PVTT) that examines general attention via sustained attention reaction times by reacting to a visual stimulus using a keyboard [32,34,35,40,41]. Participants practiced each test during familiarization sessions three times to establish test reliability and minimize a learning effect. During each testing session, tests were administered in randomized order for each testing session took about 30–35 min to complete.

### 3.4. Light Tracking Reaction Test

The NeuroTracker Pro on-site system (NeuroTracker, Montreal, QC, Canada) light-tracking reaction performance test in 3-dimension mode was administered to assess perceptual-cognitive skills, as previously described [26]. The system was operated using a Zephyrus GX501 gaming laptop (AsusTek Computer Inc., Taipei, Taiwan) with a wireless Logitech G PRO gaming mouse (Logitech Europe S.A., Lausanne, CHE). During this test, participants wore BOBLOV JX-30 3D DLP-link active shutter glasses (Shenzhen Technology Co., Ltd., Shenzhen, Guangdong, China). Participants played three CORE sessions each, including 20 × 8-s trials (i.e., 3 × 6 min sessions). During each trial, 6–8 yellow balls were displayed on the wall using a 3D projector (DLP projector, Optoma Corp., New Taipei City, Taiwan). Four balls were identified as targets to be tracked (identification phase). The objects then began moving within the 3D environment at a designated speed for 8 s (removal phase), at which point the motion stopped. Using their gaming mouse, participants had to identify which of the newly located balls were the designated targets (stoppage phase). The system then gave feedback about accuracy (feedback phase). The ball’s motion speed increased or decreased after every trial, in multiples of 68 cm/second (i.e., 1 = 68 cm/s; 2 = 136 cm/s; and so on), depending on the participant’s ability to correctly identify targets. Trial duration, start speed, percentage of targets correctly identified, and the total score were evaluated to assess performance. We previously reported a test-retest Cv of identifying correct targets from this test of 6.5% [26].

### 3.5. Gaming Performance Assessment

Scores were taken from five videogames (i.e., Call of Duty, Super Smash Brothers, Overwatch, League of Legends, and Valorant) where participants played as many rounds/matches as possible of their chosen game for approximately 1 h. Wait times between matches (queue time) were accounted for to maintain consistency between sessions. Of the five videogames, scores collected from Call of Duty and Super Smash Brothers were from varying gameplay modes, 7 and 3, respectively. All gaming scores were normalized using “Min-Max Scaling”, which rescales data points between 0 and 1, using the lowest and highest values, establishing a relative range. Each variable (e.g., kills, deaths, etc.) was recalculated (Xnew = (X − Xmin)/ (Xmax − Xmin)) and assigned a new value (Xnew), using the highest (Xmax) and lowest (Xmin) scores for that game/mode [42]. For every round/match of gameplay, the scores were rescaled and then averaged to create a single “round performance score.” The round performance scores, within the same testing session, were then averaged and used as a “performance score” for the whole session. Normalization of the gaming scores yielded relative values, allowing for comparison across game modes, titles, and platforms.

### 3.6. Profile of Mood States

The 65-item Profile of Mood States (POMS) questionnaire was used to evaluate changes in mood states [43,44]. Participants were given a list of words and asked to rate how well each described their mood over the past week on a scale where 0 represents not at all, 1 represents a little, 2 represents moderately, 3 represents quite a bit, and 4 represents extremely. Responses are categorized into six domains (i.e., tension, depression, anger, fatigue, vigor, and confusion). A total mood disturbance score is also calculated by adding scores from tension, depression, anger, fatigue, and confusion and subtracting the vigor score [44]. The POMS has been used extensively to assess mood states in various healthy and clinical populations [43,44,45], including gamers [25].

### 3.7. Ratings of Fatigue and Eye Irritability

Participants were asked to rate general fatigue and eye irritability using a visual analogical scale (VAS) where 0 represented no fatigue/eye irritability and 10 represented maximal fatigue/eye irritability.

### 3.8. Side Effects and Sleep Quality

The symptoms of dizziness, tachycardia, heart palpitations, shortness of breath, blurred vision, and nervousness were assessed using separate Likert-type scales for frequency and severity, respectively, where 0 represented none; 1 represented 1–2 per week or minimal; 2 represented 3–4 per week or slight; 3 represented 5–6 per week or moderate; 4 represented 7–8 per week or severe; and 5 represented ≥9 per week or very severe. Participants also reported any other side effects they may have experienced in response to taking the assigned groups. Reliability in answering these side effects questions in our lab yielded means Cv’s ranging from 1.2 to 2.6% [46]. Participants completed a sleep quality questionnaire that included questions about bedtime, length of time to fall asleep, waking time, hours of sleep per night, whether they had trouble sleeping, and to rate their overall sleep quality. Participants were asked to rate these items as very bad, fairly bad, fairly good, or very good within the past 48 h. They were also asked to rate their enthusiasm in getting things done as not a problem at all, a very slight problem, somewhat of a problem, or a very big problem. Finally, questions were asked regarding how their partner (if any) perceived their sleep quality and if any other restlessness was experienced.

### 3.9. Statistical Analysis

Data were analyzed by the IBM^®^ Version 29 SPSS^®^ statistical analysis software (IBM Corp., Armonk, NY, USA). The study sample size was selected based on our previous work in this area [26,33,47,48] assuming an anticipated improvement of 5% with a power of 80% in primary outcome cognitive function-related variables. The sample size also provided sufficient power to assess clinically significant side effects [26,47,48,49,50]. Participants meeting eligibility criteria were stratified into three groups based on body weight and sex and then randomized into groups in a counterbalanced manner. A general linear model (GLM) multivariate and univariate analyses with repeated measures of time and groups were used to analyze the data. Sphericity was assessed using Mauchly’s test, while skewness and kurtosis statistics assessed normality. The Wilks’ Lambda and Greenhouse–Geisser univariate correction tests were used to assess time and group x time interaction effects. A type I error (*p*-level) probability was set at 0.05 or less. Statistical tendencies were noted when *p*-values >0.05 to <0.10 were observed. Pairwise differences were assessed using Fisher’s least significant difference adjustment statistic. The clinical significance of the findings was evaluated by assessing mean changes with 95% confidence intervals (CI). Means and 95% CIs above or below baseline were considered statistically and clinically significant [51]. Data are means ± standard deviations (SD) or mean changes from baseline (mean change (LL, UL)). Partial Eta squared (η_p_^2^) values were used to assess effect size, where 0.01 indicated a small effect, 0.06 indicated a medium effect, and 0.14 indicated a large effect size [52]. Missing data (1.27% of 55,056 data points) were replaced with series means, the most common frequency data number modified for group and sex. Missing gaming data were not replaced.

## 4. Results

### 4.1. Demographic Data

Table S1a shows participant demographic data. A total of 10 women and 51 men completed the study. Participants were 21.7 ± 4.1 years, 173.4 ± 8.2 cm, 73 ± 13 kg, and 24.2 ± 3.6 kg/m^2^. Participants also had a resting heart rate of 76.1 ± 15.4 bpm, a systolic blood pressure of 121.6 ± 11.8 mmHg, and a diastolic blood pressure of 74.8 ± 7.5 mmHg. Sex differences were noted in height, weight, and resting heart rate. Table S1b shows the video games participants played in each group during the visits, along with a brief description and links to game websites that provide more details about the games.

### 4.2. PEBL Cognitive Function Assessment

#### 4.2.1. Berg-Wisconsin Card Sorting Test

**Figure 4:**
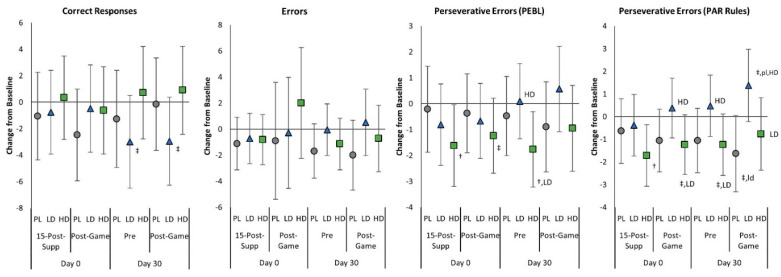
Berg-Wisconsin Card Sorting Test mean changes with 95% confidence intervals from Day 0, pre-game (Pre) values for the placebo (PL), low-dose (LD), and high-dose (HD) groups. ^†^ represents *p* < 0.05 from Pre values while ^‡^ represents *p* > 0.05 to *p* < 0.10 effect. 15-min-Post-Supp represents data obtained 15 min after supplementation, and Post-Game represents data obtained after competitively playing video games for 60 min. Group differences (*p* < 0.05) are shown as differences from placebo (pl), low-dose (ld), and high-dose (hd) groups, while statistical tendencies (*p* > 0.05 to *p* < 0.10) are shown in large cases (PL, LD, HD).

Table S2 presents the BCST results observed. Analysis of BCST data results revealed no significant overall time (*p* = 0.730, η_p_^2^ = 0.013, small effect) or group × time effects (*p* = 0.446, η_p_^2^ = 0.034, small effect). Univariate analysis revealed no significant group × time effects in correct responses, errors, or perseverative errors. However, perseverative errors with PAR rules (revised scoring method) tended to differ with a moderate effect size (*p* = 0.088, η_p_^2^ = 0.058, medium effect). Both perseverative errors significantly decreased following acute ingestion of HD and remained lower than baseline after 30 days of supplementation. After 30 days of supplementation, the PL group had significantly fewer perseverative errors (PAR rules) than the LD group and the PL and HD groups had more correct responses after gaming. Similarly, analysis of changes from baseline with 95% CIs (Figure 4) revealed that Day 0 Pre and Post-Game correct responses tended to be lower than baseline in the LD group with no significant differences observed among groups. Acute HD ingestion significantly decreased perseverative errors (PEBL and PAR rules) from baseline, with Post-Game values tending to be lower than the LD group. However, it should be noted that the HD group had non-significantly higher baseline levels which may account for some of these differences. After 30 days of supplementation, the LD group tended to have fewer correct Pre and Post-Game responses, while there was evidence that perseverative errors (PEBL and PAR rules) were significantly reduced from baseline with HD ingestion with perseverative errors (PAR rules) tending to be lower than the LD group but not the PL group.

#### 4.2.2. Go/No-Go Task Test

**Figure 5:**
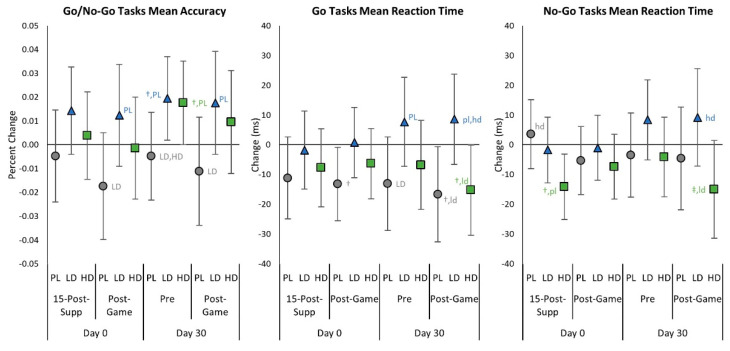
Go/No-Go Task test mean changes with 95% confidence intervals from Day 0, pre-game (Pre) values for the placebo (PL), low-dose (LD), and high-dose (HD) groups. ^†^ represents *p* < 0.05 from Pre values while ^‡^ represents *p* > 0.05 to *p* < 0.10 effect. 15-min-Post-Supp represents data obtained 15 min after supplementation, and Post-Game represents data obtained after competitively playing video games for 60 min. Group differences (*p* < 0.05) are shown as differences from placebo (pl), low-dose (ld), and high-dose (hd) groups, while statistical tendencies (*p* > 0.05 to *p* < 0.10) are shown in large cases (PL, LD, HD).

Table S3 presents Go/No-Go Task test results. GLM multivariate analysis revealed no significant time (*p* = 0.348, η_p_^2^ = 0.019, small effect; *p* = 0.522, η_p_^2^ = 0.016, small effect) or group × time interaction effects (*p* = 0.109, η_p_^2^ = 0.044, small to medium effect; *p* = 0.389, η_p_^2^ = 0.035, small effect) in Go or No-Go Task variables, respectively. Likewise, no significant time or group × time interaction effects were seen from GLM univariate analysis. Figure 5 shows the mean changes from baseline with 95% CIs. On Day 0, No-Go Tasks mean reaction time was faster at 15-Post-Supp with HD, suggesting some improvement within 15 min of supplementation. Day 0 Post-Game mean accuracy was lower than baseline in the PL group but Go and No-Go Tasks mean reaction times were faster. Conversely, HD ingestion promoted faster No-Go reaction times without affecting accuracy. After 30 days of supplementation, there was evidence that Pre mean accuracy was higher than the baseline with LD and that mean accuracy in the LD and HD groups tended to be higher than PL. That said, reaction times were slower with LD, whereas HD reaction times were comparable to or faster than PL. Day 30 Post-Game accuracy and reaction times were best with HD ingestion.

#### 4.2.3. Sternberg Task Test

**Figure 6:**
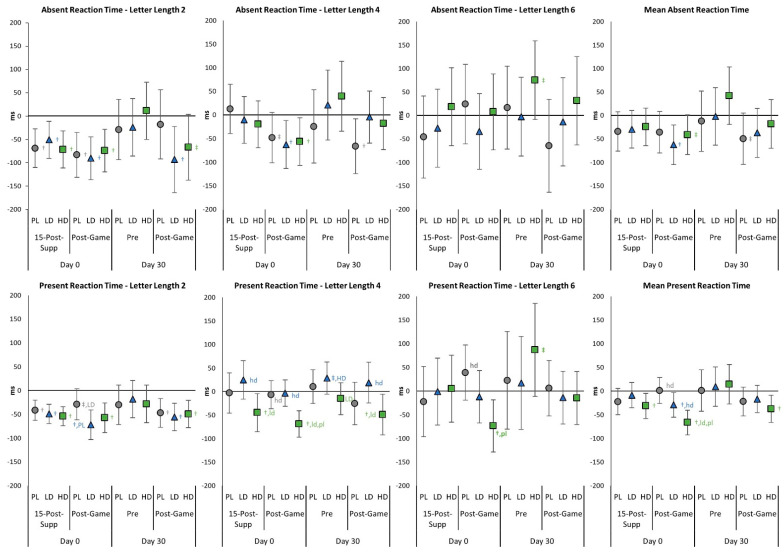
Sternberg Task Test mean Absent and Present Reaction Time changes with 95% confidence intervals from Day 0, pre-game (Pre) values for the placebo (PL), low-dose (LD), and high-dose (HD) groups. ^†^ represents *p* < 0.05 from Pre values while ^‡^ represents *p* > 0.05 to *p* < 0.10 effect. 15-min-Post-Supp represents data obtained 15 min after supplementation, and Post-Game represents data obtained after competitively playing video games for 60 min. Group differences (*p* < 0.05) are shown as differences from placebo (pl), low-dose (ld), and high-dose (hd) groups, while statistical tendencies (*p* > 0.05 to *p* < 0.10) are shown in large cases (PL, LD, HD).

Table S4 shows the STT results. Multivariate analysis revealed a significant time effect (*p* < 0.001, η_p_^2^ = 0.097, medium effect) with no significant group × time interaction effects observed (*p* = 0.114, η_p_^2^ = 0.041, small effect). Univariate analysis revealed significant time effects in several letter length reaction time scores with some groups experiencing differential changes over time in Day 0 or Day 30 tests. However, while some moderate effect sizes were seen, no significant group × time effects were observed. Figure 6 presents mean changes observed from baseline in Absent and Present reaction times. Acute HD ingestion saw faster Letter Length 4 and Mean Present Reaction Times from baseline. Post-gaming Letter Length 4 and Mean Absent Reaction Times were faster than baseline in the LD and HD groups. Additionally, on Day 0, the HD group Post-Game Letter Lengths 2, 4, 6, and Mean Present Reaction Times were faster than baseline and PL and/or LD groups. In fact, analysis of Post-Game Present Reaction Time at increasing letter lengths revealed a significant interaction effect (*p* = 0.046, η_p_^2^ = 0.065, medium effect), with pairwise comparisons showing the HD group was the only group to maintain Present Reaction Times as task difficulty increased. Present Reaction Times in the HD group were also significantly faster than PL at the letter lengths 4 and 6 after acute supplementation (see Figure S1). After 30 days, Letter Length 6 Absent and Present Reaction Times tended to be slower in the HD group than LD and PL. However, Post-Game Letter Length 4 values and Mean Present Reaction Times were faster in the HD group. Thus, analysis of changes from baseline suggests that acute and 30-day LD and HD supplementation may improve reaction times, potentially leading to better gaming performance.

#### 4.2.4. Psychomotor Vigilance Task Test

**Figure 7:**
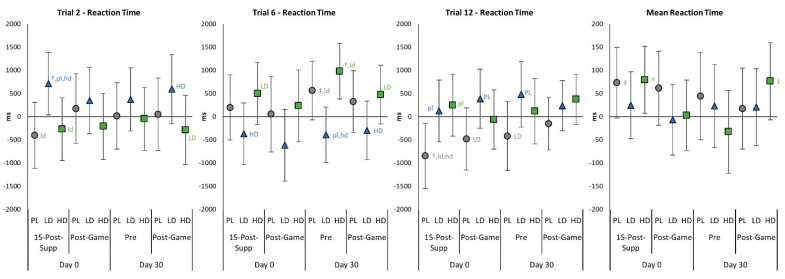
Psychomotor Vigilance Task Test changes with 95% confidence intervals from Day 0, pre-game (Pre) values for the placebo (PL), low-dose (LD), and high-dose (HD) groups. ^†^ represents *p* < 0.05 from Pre values while ^‡^ represents *p* > 0.05 to p < 0.10 effect. 15-min-Post-Supp represents data obtained 15 min after supplementation, and Post-Game represents data obtained after competitively playing video games for 60 min. Group differences (*p* < 0.05) are shown as differences from placebo (pl), low-dose (ld), and high-dose (hd) groups, while statistical tendencies (*p* > 0.05 to *p* < 0.10) are shown in large cases (PL, LD, HD).

Table S5 shows the results of the PVTT test. Multivariate analysis revealed no significant time (*p* = 0.541, η_p_^2^ = 0.045, small effect) or group × time interaction effects (*p* = 0.755, η_p_^2^ = 0.042, small effect). Univariate analysis revealed no significant time or group × time effects, although some moderate effect sizes were observed. However, pairwise comparisons revealed that reaction times in the LD group were faster than PL at several points, particularly during the beginning of the 20 trials evaluated. Figure 7 shows mean changes in PVTT-related variables with 95% CIs. Acute supplementation had variable effects on psychomotor vigilance at 15-Post-Supp and following 60-min of gaming (Post-Game) by dose amount with no significant differences compared to PL. After 30 days of supplementation, Pre Trial 6 reaction time was faster with LD supplementation compared to PL and HD yet slower than PL in Trial 12. Thus, while some differences were seen, responses were inconsistent and generally not significantly different than PL responses.

### 4.3. Light Reaction Test

**Figure 8:**
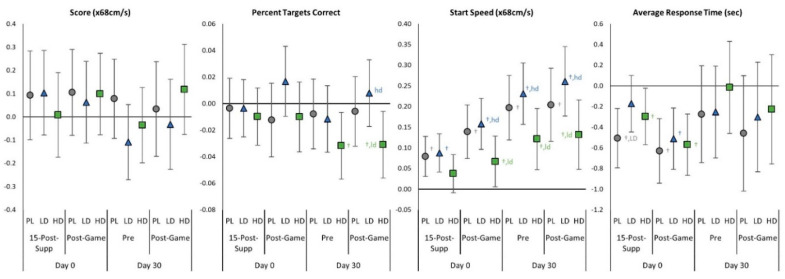
Light reaction test changes with 95% confidence intervals from Day 0, pre-game (Pre) values for the placebo (PL), low-dose (LD), and high-dose (HD) groups. ^†^ represents *p* < 0.05 from Pre values while ^‡^ represents *p* > 0.05 to *p* < 0.10 effect. 15-min-Post-Supp represents data obtained 15-min after supplementation and Post-Game represents data obtained after competitively playing video games for 60 min. Group differences (*p* < 0.05) are shown as differences from placebo (pl), low-dose (ld), and high-dose (hd) groups, while statistical tendencies (*p* > 0.05 to *p* < 0.10) are shown in large cases (PL, LD, HD).

Table S6 shows the results of the light reaction test. Multivariate analysis revealed a significant time effect (*p* < 0.001, η_p_^2^ = 0.159, large effect) with no significant group × time interaction effects observed (*p* = 0.531, η_p_^2^ = 0.032, small effect). No significant group × time effects were observed with univariate analysis either. Start speed increased in all groups over time, suggesting improved performance. Analysis of mean changes with 95% Cis (Figure 8) revealed some evidence of differences among groups in start speed and percentage of correct targets. On Day 0, the start speed was increased from baseline at 15-Post-Supp in the PL and LD groups but not HD. Post-Game start speed increased in all groups with LD responses higher than HD, suggesting an improved performance, although it was not different from PL. No significant differences were seen on Day 0 in 15-Post-Supp or Post-Game percent targets correct or score among groups. After 30 days of supplementation, Pre gaming start speed was better maintained with LD ingestion compared to HD, while the percentage targets correct was decreased in the HD group compared to baseline values. Post-Game start speed was higher than baseline with PL, LD, and HD. Post-Game start speed and percentage targets correct were higher in the LD group compared to the HD, although not significantly different from PL.

### 4.4. Gaming Performance

**Figure 9:**
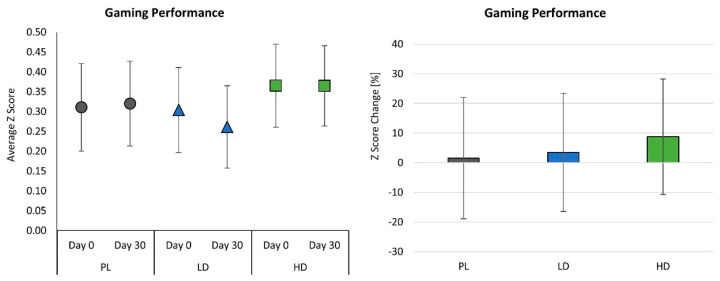
Mean gaming performance Z scores and percent change from baseline with 95% confidence intervals in gaming performance.

Table S7 presents normalized gaming performance results expressed as Z scores on 57 participants with measurable game scores that could be statistically analyzed (PL = 18, LD = 19, HD = 20). No significant time (*p* = 0.536, η_p_^2^ = 0.007, small effect) or group × time effects (*p* = 0.468, η_p_^2^ = 0.028, small effect) were observed among Day 0 and Day 30 performance scores (Figure 9). One-way analysis of variance of mean changes from baseline in gaming revealed no significant differences among groups (PL 0.0091 ± 0.087; LD −0.0428 ± 0.174; HD −0.0002 ± 0.133, *p* = 0.468, η_p_^2^ = 0.028, small effect) or between PL scores and LD (0.052 (−0.038, 0.142), *p* = 0.254) and HD (0.009 (−0.080, 0.098), *p* = 0.836) values. Likewise, no significant differences were observed among groups in percent changes in Z-scores (PL 1.56 ± 28.9; LD 3.53 ± 52.4; HD 8.76 ± 44.7%, *p* = 0.868, η_p_^2^ = 0.005, small effect) or between PL scores and LD (−1.96% (−30.5, 26.6), *p* = 0.891) and HD (−7.20% (−35.4, 21.0) *p* = 0.611) values. These findings indicate that while mean percentage changes in performance appeared more favorable with LD and HD, they were not significant due to large variability. Therefore, performance was consistent among groups.

### 4.5. Mood State, Fatigue, and Eye Irritability Ratings

**Figure 10:**
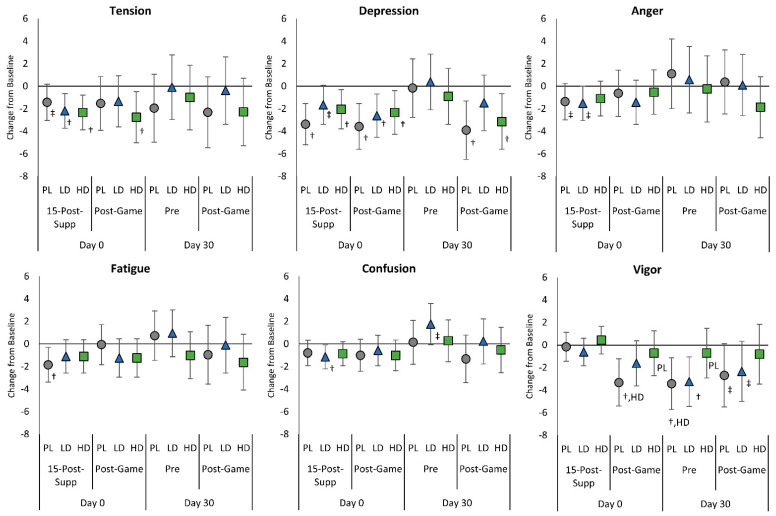
Profile of Mood State domain changes from baseline with 95% confidence intervals from Day 0, pre-game (Pre) values for the placebo (PL), low-dose (LD), and high-dose (HD) groups. ^†^ represents *p* < 0.05 from Pre values while ^‡^ represents *p* > 0.05 to *p* < 0.10 effect. 15-min-Post-Supp represents data obtained 15-min after supplementation and Post-Game represents data obtained after competitively playing video games for 60 min. Group differences (*p* < 0.05) are shown as differences from placebo (pl), low-dose (ld), and high-dose (hd) groups, while statistical tendencies (*p* > 0.05 to *p* < 0.10) are shown in large case (PL, LD, HD).

Table S8a presents the results of the Profile of Mood States (POMS) assessment, while Table S8b shows individual responses to the POMS questionnaire. Overall multivariate analysis revealed a significant time effect (*p* < 0.001, η_p_^2^ = 0.083, medium effect) with no significant group × time interaction effects observed (*p* = 0.836, η_p_^2^ = 0.027, small effect). Similarly, no significant group x time effects were observed with univariate analysis. However, pairwise comparisons indicated that tension, confusion, and vigor scores with the LD group were generally lower than PL responses at several points. Conversely, changes in vigor scores were generally higher in the HD group. However, no significant differences were seen in the total mood state disturbance score. Figure 10 shows mean changes in POMS domain scores with 95% CIs. Acute supplementation Post-Game vigor scores significantly declined in the PL and LD groups from baseline but not in the HD group. After 30 days of supplementation, Pre and Post-Game vigor scores were also better maintained with HD supplementation than PL and LD. Analysis of individual items (Table S8b) provides some evidence that ratings for tense, unhappy, sorry for things done, shaky, peeved, on edge, panicky, discouraged, nervous, miserable, anxious, sluggish, weary, bewildered, and terrified were lower in the LD and/or HD groups than PL. In comparison, ratings of lively, fatigued, cheerful, and ready-to-fight were higher in the LD and/or HD groups than in PL at various time points. No significant differences were seen in ratings of eye fatigue or eye irritation among groups (see Table S9). However, after 30 days of supplementation, participants taking the HD supplement did report a significant reduction in eye irritation.

### 4.6. Safety and Sleep Assessments

Table S10a,b present the frequency and severity of common side effects, respectively. There were no significant interactions among groups. Finally, Table S11a,b show the results of the sleep quality assessment. No significant differences were observed in sleep-related variables. These findings suggest that the supplements were well tolerated.

## 5. Discussion

Video gaming and esports have become an increasingly popular, highly competitive, and multibillion-dollar industry [6]. To succeed at gaming, players must have quick reaction skills and high cognitive abilities to assess and respond to game challenges [6]. The ability to be alert, focus, maintain attention, make quick decisions, and navigate through complex tasks is essential [6]. Most esport tournaments require playing multiple competitive games over several days. Consequently, identifying strategies to improve cognitive function, delay mental fatigue, and maintain psychomotor skills is imperative for success. This study examined whether ingesting a microalgae extract containing fucoxanthin with a natural source of caffeine (i.e., guarana) affects cognitive function before and/or following competitive gaming in experienced gamers compared to ingesting a natural source of caffeine alone. Present findings provide evidence that acute and 30-day ingestion of microalgae extract containing *PT* with Guarana containing the amount of caffeine found in a serving of espresso may improve reaction times, reasoning, learning, executive control, attention shifting (cognitive flexibility), and impulsiveness. While some effects were observed after acute ingestion, the greatest impact appeared after 30 days of supplementation, with some beneficial effects seen in both the LD and HD groups. Moreover, this combination of ingredients delivered at low and high doses may support various aspects of mood after acute and 30 days of supplementation without side effects.

An emerging area of performance enhancement is identifying nutritional strategies to help individuals improve and/or maintain cognitive function during work and/or competition. Several studies have evaluated the effects of nutritional supplementation on cognitive function and/or video game performance. For example, Cereda and coworkers [53] reported that consuming an energy drink containing a mixture of sugars, vitamins, and carnitine for 3 weeks improved the time and number of trials needed to complete video games. Kennedy and colleagues [12] reported that a small amount of guarana (75 mg containing about 9 mg of caffeine) increased attention tasks and performance speed compared to *Panax ginseng*. Additionally, ingesting guarana (222 mg with 40 mg of caffeine) with a multivitamin and mineral blend improved task performance compared to placebo [11]. Similarly, Doma and colleagues [54] reported that ingesting a coffee and cherry extract with phosphatidylserine enhanced memory, focus, concentration, accuracy, and learning using the Computerized Mental Performance Assessment System (COMPASS) battery of cognitive tests in healthy adults who self-reported memory problems. However, supplementation did not affect everyday memory questionnaire (EMQ) responses, or the Go/No-Go test used in the present study. Cardona and coworkers [55] reported that eight weeks of multi-probiotic supplementation reduced errors of omission in Go trials (indicating an improvement in attention), with no impact on memory in patients with fibromyalgia. Moreover, Kackley and associates [56] reported that ingesting ketogenic salts may attenuate negative aspects of mood associated with diet intervention. Our group has also reported that acute and one-week ingestion of paraxanthine (the compound that caffeine is primarily converted into after ingestion) [33,57], as well as ashwagandha [58], appears to improve short-term memory, reaction times, and/or errors in healthy individuals. These findings are consistent with studies reporting that various nutrients (with and without caffeine) may influence cognitive function. Additionally, combining various nootropic nutrients may provide some additive and/or synergistic benefit [7,9,10,14,27,33,47,54,55,57,59,60].

Regarding gaming performance, Kalman et al. [27] found that ingesting 1500 mg of inositol-stabilized arginine silicate (ASI) improved cognitive function, flexibility, processing, and executive function. Similarly, Tartar and associates [25] reported that ingestion of 1500 mg of ASI + 100 mg of additional inositol for 1 and 7 days prior to playing video games for 60 min in gamers improved accuracy, decision making, and reaction time in cognitive and gaming tests. Our group also reported that ingesting 1500 mg of ASI with 100 mg of inositol before playing video games enhanced some measures of working memory, reasoning, and concentration in experienced gamers [26]. Bloomer et al. [28] reported that pre-gaming ingestion of a natural supplement containing caffeine (270 mg) with polyphenolic antioxidants had no significant effects on gaming or cognitive performance compared to caffeine or a placebo. However, some statistical trends related to improved gaming performance (i.e., kills/matches) were observed. Finally, Thomas and coworkers [8] reported that consumption of an energy drink containing 150 mg of caffeine with l-carnitine, l-theanine, phosphatidylserine, choline, and nicotinamide adenine dinucleotide (reduced NADH) with some vitamins and minerals prior to gaming had no significant effects on several cognitive function tests including the Go/No-Go task test. Consequently, there is evidence that ingestion of several nutrients can affect cognition, attention, and/or psychomotor performance.

Present findings are consistent with our previous findings [26] that the Go/No-Go Task and STT tests seem more sensitive to cognitive performance than the BCST and PVTT assessments. In particular, while there was some evidence that acute and 30 days of LD and HD supplementation positively affected markers of cognitive function, HD ingestion promoted faster mean reaction times within 15 min of ingestion in the Go/No-Go Task test. Additionally, 30 days of HD supplementation had faster reaction times in Go and No-Go tasks. These findings suggest an improvement in attention and response inhibition [61]. Improvements in Go/No-Go reaction times were similar to findings we previously reported with the caffeine metabolite paraxanthine [33,57]. Labente and Nielson [62] recently noted that while the Go/No-Go task test can serve as a valuable tool to assess the effects of food and nutritional interventions on cognition, it is vital to standardize experimental conditions to ensure valid results. In the present study, we performed several practice trials during the familiarization session to normalize responses. Participants also replicated their diet four days before testing sessions; refrained from ingesting supplements and energy drinks; tested at the same time of day; prepared for testing sessions like they would a competitive event; and continued playing their game at normal weekly amounts between testing sessions. Thus, incorporating these types of controls may have improved the ability to identify differences with the Go/No-Go test. Results of the STT assessment revealed that on Day 0, participants in the HD group could maintain Post-Game Present Reaction Times as task difficulty increased from 2 to 4 and 6 letters. In contrast, reaction times slowed in the PL group. While this trend was not seen after 30 days of supplementation, the HD group was the only group that observed significantly faster Post-Game Mean Present Reaction times from baseline. The microalgae-based ingredient evaluated in this study is the first natural ingredient we are aware of that showed some cognitive function improvement in gamers. Other natural blends of caffeine and polyphenols studied were seemingly less impressive [28]. Overall, this study’s findings support contentions that esport gamers may benefit from acute and/or chronic dietary supplementation of nutrients that affect cognitive function.

Nutritional strategies intended to enhance cognitive function have focused on increasing blood flow [25,26,27,63], energy availability [64,65], and/or neurotransmission in the brain and reducing oxidative stress and inflammation [65,66,67,68,69]. The *PT* extract used in the present study contains carotenoid fucoxanthin that can bypass the blood–brain barrier. They have been reported to possess neuroprotective, anti-inflammatory, and antioxidant properties [18,19,20]. For example, fucoxanthin may (1) help prevent hydrogen peroxide (H_2_O_2_)-induced DNA damage and promote antioxidant defense in lipopolysaccharide (LPS)-activated Bv2 microglia by activating Nrf2 signaling pathway and cell survival through activating cAMP-dependent protein kinase response; (2) have anticholinesterase functionality which may increase neurotransmitters’ actions and synaptic transmission [70] which could help improve reaction time to make quick decisions; (3) increase brain-derived neurotrophic factor (BDNF), which is related to short-term memory improvement [71]; and (4) exhibit neurite outgrowth activity which may improve learning ability by increasing neuronal networks [70,72]. Other molecules found in the microalgae extract of *PT* such as phycoprostans can affect immature brain cells such as oligodendrocyte progenitors and increase the speed of propagation of nerve impulses by protecting neurons against oxidant injury and promoting myelination [23]. Unpublished proof-of-concept data in the D-GAL mice model in the review found that adding PT to feed at a human dose equivalent to 120 mg/day significantly improved short-term and spatial working memory (i.e., Y-Maze and Morris Water Maze tests). Wang et al. [19] also showed that the *PT* extract significantly decreases brain and blood inflammation (IL-6) and oxidative stress (lipid peroxidation), which are mechanisms largely involved in cognitive performance. Moreover, microalgae varieties such as *PT* also contain LC-PUFAs and EPA that have been reported to reduce neuroinflammation and oxidative stress, thereby providing neuroprotection [15,16,17,21,22]. Finally, since guarana contains caffeine, it may improve cognitive performance [11,12,13,73] and help maintain focus and mental energy by stimulating the central nervous system (e.g., blocking adenosine activity) [60]. Consequently, there is a good theoretical rationale to determine the effects of co-ingestion PT with a natural source of caffeine.

The strengths of this study were that it evaluated the effects of low and high-dose supplementation with *PT* extract combined with guarana on cognitive function in a fairly large cohort of experienced gamers using their most familiar game. As gamers possess better cognitive functions than those who do not play video games [2,3,5], they represent an interesting research model that may apply to other populations. Additionally, cognitive function was assessed using a battery of tests to better understand how acute and 30 days of *PT* extract combined with guarana supplementation may influence memory, reaction times, reasoning, and gaming performance. Moreover, this study assessed how this nutritional strategy might affect mood states, stimulant-related side effects, and sleep indices.

Limitations included assessing the performance of several different game versions and play modes, which made the interpretation of performance more difficult, given the differences among games and their scoring parameters. There also could have been differences in energy, mental focus, cognitive and psychomotor challenges, and fatigue associated with playing different games. These differences may have increased variability among participants in cognitive function assessments. Furthermore, despite practicing the cognitive function tests, normal variability in performing cognitive function tests may have increased type II error. Assessing a cohort primarily comprised of men was also a limitation.

Future research should select one game to evaluate or simulate an esport tournament and consider monitoring weekly adaptations during gaming practice sessions on performance adaptations. Additional research should also assess the effects of acute and chronic *PT* with guarana supplementation in students, athletes, and office workers to promote attention, focus, and/or memory. For example, students often stay up for hours the night before taking exams. Driving long distances requires alertness, quick decisions, good reaction skills, and decision making. Finally, military personnel, tactical athletes, and first responders must make decisions under duress and/or in challenging conditions and environments [1]. These populations might benefit from taking nutrients affecting cognition and/or psychomotor performance. Finally, additional work could examine potential pre-clinical applications on mood state, depression, anxiety, and eye fatigue.

## 6. Conclusions

The results provide some evidence that acute and 30-day ingestion of microalgae extract from *PT* combined with guarana may improve reasoning, learning, executive control, attention shifting, and impulsiveness. While some acute effects were noted after 15 min of ingestion and after gaming, the greatest impact appeared after 30 days with some benefits seen at both low and high doses. Moreover, there was evidence that microalgae extract from *PT* combined with guarana may support mood domains after acute and 30 days of supplementation at both doses studied. While more research is needed to better understand potential mechanisms of actions, these findings support contentions that ingesting *PT* microalgae extract with a natural source of caffeine before gaming and days prior to competition may provide some benefit to reaction times, cognitive function, and gaming performance. Finally, considering that gamers are an interesting research model to study due to high basal cognitive function levels [2,3,5], results observed in this study open application perspectives in other populations such as athletes, students, and people with stress, anxiety, or depression issues.

## Data Availability

Data and statistical analyses are available for non-commercial scientific inquiry and/or educational if request and use do not violate IRB restrictions and/or research agreement terms.

## Supplementary Materials

The following supplemental materials are available online at https://www.mdpi.com/article/10.3390/nu15081918/s1, Table S1: (a) Demographic data, (b) Videogames played, Table S2: Berg-Wisconsin Card Sorting Task results, Table S3: Go/No-Go Task Test results, Table S4: Sternberg Task Tests reaction time data, Table S5: Psychomotor Vigilance Task Test results, Table S6: Light reaction test results, Table S7: Gaming Performance, Table S8a: Profile of Mood States results, Table S8b: POMS item analysis, Table S9: Fatigue and Eye Irritability rating results, Table S10: (a) Frequency of Side Effects, (b) Severity of Side Effects, Table S11: (a) Sleep quality questionnaire results, (b) Sleep quality questionnaire categorical results. Figure S1: Sternberg task test Post-game Present Reaction Times at increasing letter lengths. Data are mean changes from baseline with 95% confidence intervals for the placebo (PL), low-dose (LD), and high-dose (HD) groups. ^†^ represents *p* < 0.05 from Pre values while ^‡^ represents *p* > 0.05 to *p* < 0.10 effect. Group differences (*p* > 0.05) are shown as differences from placebo (pl), low-dose (ld), and high-dose (hd) groups while statistical tendencies (*p* > 0.05 to *p* < 0.10) are shown in large cases (PL, LD, HD).
